# Real-World Evidence in Glioblastoma: Stupp's Regimen After a Decade

**DOI:** 10.3389/fonc.2020.00840

**Published:** 2020-07-03

**Authors:** Radek Lakomy, Tomas Kazda, Iveta Selingerova, Alexandr Poprach, Petr Pospisil, Renata Belanova, Pavel Fadrus, Vaclav Vybihal, Martin Smrcka, Radim Jancalek, Ludmila Hynkova, Katarina Muckova, Michal Hendrych, Jiri Sana, Ondrej Slaby, Pavel Slampa

**Affiliations:** ^1^Department of Comprehensive Cancer Care, Masaryk Memorial Cancer Institute, Brno, Czechia; ^2^Department of Comprehensive Cancer Care, Faculty of Medicine, Masaryk University, Brno, Czechia; ^3^Department of Radiation Oncology, Masaryk Memorial Cancer Institute, Brno, Czechia; ^4^Department of Radiation Oncology, Faculty of Medicine, Masaryk University, Brno, Czechia; ^5^Research Center for Applied Molecular Oncology, Masaryk Memorial Cancer Institute, Brno, Czechia; ^6^Department of Radiology, Masaryk Memorial Cancer Institute, Brno, Czechia; ^7^Faculty of Medicine, Masaryk University, Brno, Czechia; ^8^Department of Neurosurgery, University Hospital Brno and Faculty of Medicine, Masaryk University, Brno, Czechia; ^9^Department of Neurosurgery, St. Anne's University Hospital Brno and Faculty of Medicine, Masaryk University, Brno, Czechia; ^10^Department of Pathology, University Hospital Brno and Faculty of Medicine, Masaryk University, Brno, Czechia; ^11^First Department of Pathology, St. Anne's University Hospital and Faculty of Medicine, Masaryk University, Brno, Czechia

**Keywords:** glioblastoma, chemotherapy, radiotherapy, rapid early progression, overall survival, real-world evidence

## Abstract

The aim of this retrospective study is to provide real-world evidence in glioblastoma treatment and to compare overall survival after Stupp's regimen treatment today and a decade ago. A current consecutive cohort of histologically confirmed glioblastoma irradiated from 1/2014 to 12/2017 in our cancer center was compared with an already published historical control of patients treated in 1/2003–12/2009. A total of new 155 patients was analyzed, median age 60.9 years, 61% men, 58 patients (37%) underwent gross total tumor resection. Stupp's regimen was indicated in 90 patients (58%), 65 patients (42%) underwent radiotherapy alone. Median progression-free survival in Stupp's regimen cohort was 6.7 months, median OS 16.0 months, and 2-year OS 30.7%. OS was longer if patients were able to finish at least three cycles of adjuvant chemotherapy (median 23.3 months and 43.9% of patients lived at 2 years after surgery). Rapid early progression prior to radiotherapy was a negative prognostic factor with HR 1.87 (*p* = 0.007). The interval between surgery and the start of radiotherapy (median 6.7 weeks) was not prognostically significant (*p* = 0.825). The median OS in the current cohort was about 2 months longer than in the historical control group treated 10 years ago (16 vs. 13.8 months) using the same Stupp's regimen. Taking into account differences in patient's characteristics between current and historical cohorts, age, extent of resection, and ECOG patient performance status adjusted HR (Stupp's regimen vs. RT alone) for OS was determined as 0.45 (*p* = 0.002).

## Introduction

Despite intensive multimodal treatment of glioblastoma consisting of maximal safe resection followed by combined chemoradiotherapy (well-known Stupp's regimen), ultimately all patients develop tumor recurrence and subsequently die for further glioblastoma progression (1, 2). The greatest benefit from multimodal treatment has been demonstrated in patients after macroscopic gross total resection (GTR), those under 50 years of age, with ECOG (Eastern Cooperative Oncology Group) performance status of 0–1 and the presence of promoter methylation of O6-methylguanine-DNA-methyltransferase (MGMT) gene (3–5). Currently, more approaches are considered standard of care in older people who are less tolerant to the standard Stupp's regimen and are treated by Perry's modification (3 weeks chemoradiotherapy) of by chemotherapy alone, for example (6–8).

Real-world evidence data are an increasingly important supplement to clinical and translational research. These analyses of current real-world patients treated outside controlled clinical trials may identify hidden needs as well as provide survival data for proper powering in future clinical trials. This is especially relevant in glioblastoma where no positive practice changing trial, focused on the treatment of the best prognostic glioblastoma subcohort, was published during the last 15 years despite huge advances in the understanding of glioblastoma in general (9, 10).

This single institutional retrospective study unbiased by inter-center variability aims to analyze the outcomes of consecutive glioblastoma patients irradiated in our cancer center from 1/2014 to 12/2017 and to compare their outcomes with a historical control of patients treated in 1/2003–12/2009. This control cohort with a median survival of 13 months (2-year overall survival 26%) was published in 2011 and was treated by the same Stupp's regimen as most patients from the present cohort (11, 12). Comparison of survival data in respective control arms in recently published global clinical trials (Stupp's regimen used as the standard of care in control arm) with those published in original trial by Stupp et al. (patients enrollment from 8/2000 to 3/2002) reveals remarkable improvement in survival around 5 months after the same treatment regimen (1, 10, 13, 14). We aim to describe this possible improvement also in patients treated in real-world care outside of clinical trials in single institutional report unbiased by variability associated with the inclusion of patients in many countries and institutions.

## Materials and Methods

Consecutive patients over 18 years of age with histologically proven newly diagnosed glioblastoma irradiated from 1/2014 to 12/2017 in our cancer center were eligible for this analysis approved by our institutional review board. All patients signed informed consent with the usage of their data for research purposes. All patients after glioma surgery were discussed in the multidisciplinary neurooncology tumor board and those eligible for postsurgery oncology treatment were referred to radiotherapy consultation. A subgroup of patients indicated to concurrent chemoradiation with subsequent adjuvant chemotherapy was further analyzed in detail.

Radiotherapy (RT) was performed in all patients within study cohorts. A planning CT scan for 3-dimensional RT dose calculation was utilized in all patients. Some of them underwent also planning MRI (including postcontrast T1 weighted scan with submillimeter slices) which was rigidly registered to CT scan for proper RT target definition. Individual prescription of RT dose and scheduling was guided mainly by patient's performance status and by volume, size, shape, and location of the target volume. Both standards of care approaches in target volume definitions were employed in patients eligible for treatment by Stupp's regimen—the Radiation Therapy Oncology Group (RTOG contouring approach) that defines two clinical target volumes accommodating hyperintensity at T2/FLAIR MRI in addition to T1 contrast-enhanced MRI (15) and the European Organization for Research and Treatment of Cancer (EORTC single-phase contouring approach) that defines one target utilizing mainly T1 postcontrast MRI (16). The total dose of normofractionated 60 Gy was prescribed irrespective of the used target volumes definition approach. RT was prepared employing planning system Eclipse^TM^ (Varian medical systems, Palo Alto, CA, USA) and performed on linear accelerator Varian Clinac iX or TrueBeam (Varian medical systems, Palo Alto, CA, USA).

Concurrent chemoradiotherapy and adjuvant chemotherapy were prescribed according to the original Stupp et al. (1) protocol. Temozolomide (75 mg/m^2^) was administered on days 1 through 42 with concomitant RT (60 Gy). After 4 weeks, treatment follows by the administration of temozolomide alone (150–200 mg/m^2^) on days 1–5 in six consecutive 4-week cycles or to progression. The prophylaxis against *Pneumocystis jirovecii* pneumonia was at the discretion of the treating physician.

Response to treatment was evaluated based on regular follow up MRI scanning. Progression presented already on planning MRI was considered only in patients who had available early postsurgery (within 72 h) control MRI enabling a clear definition of eventual postsurgery residuum. The first post (chemo)radiotherapy MRI was usually ordered 4–6 weeks after the last RT session, followed by regular MRI every 3 months unless clinically indicated for earlier examination. No routine RANO criteria (17) usage in daily clinical practice was employed and MRI were visually evaluated by servicing radiologist. Unclear findings were reviewed by a multidisciplinary neurooncology tumor board, mostly with a recommendation for an earlier control exam. Treatment at progression was highly individualized with options for resurgery, reirradiation, temozolomide rechallenge, palliative chemotherapy (mostly lomustine), or symptomatic treatment.

The primary objective is to evaluate the impact of clinical and laboratory factors (gender, age, extent of resection, ECOG patient status, tumor location, early tumor progression on planning MRI, MGMT methylation) and used treatment on survival parameters such as progression-free survival (PFS) and overall survival (OS). PFS was defined as the time from the date of initiation of RT to the date of relapse. Considering retrospective nature of this analysis, no strong measures according to differential diagnosis of pseudoprogression were possible to be utilized. In the cases, where progression was described by the radiologist and there was subsequent change in the treatment, we recorded date of that MRI as a date of progression. On the other hand, in the cases where there was no change in the treatment after radiologist call of possible progression and subsequent MRI did not confirm progression, we did not record the previous MRI as that with progression and the subsequent MRI were evaluated in PFS analysis. OS was defined as the time from the date of diagnosis to the date of death (from tumor cause). The last control date was considered when relapse/death was not presented. The secondary goal is to compare the current treatment results using the Stupp's regimen with the results of patients treated 10 years ago adjusted for age, extent of resection, and ECOG patient status.

Patients' characteristics of both current and historical cohorts were described using standard summary statistics i.e., median and interquartile range (IQR) for continuous variables and frequency distributions for categorical variables. The following comparison of both groups was examined with Fisher's exact test, chi-squared test, or Mann–Whitney test, as appropriate. Survival probabilities were estimated using the Kaplan–Meier method. The log-rank test was performed to compare OS and PFS between the groups. Characteristics associated with the time-to-event outcomes were evaluated using Cox models where hazard ratios (HR) and their 95% confidence interval (CI) were calculated. The proportional hazard assumption was verified based on scaled Schoenfeld residuals. The multivariable model was fitted using stepwise backward selection. All statistical analyses were performed employing R version 3.6.2 (18) and the significance level of 0.05 was considered.

## Results

A total of 155 patients was indicated to postsurgery RT. The median age was 61 years, 21% were younger 50 years, slightly higher number of men (61%). Gross total resection was achieved in 58 (37%) patients and more than 80% were in good general condition (ECOG 0–1). The other basic patients and tumor characteristics are summarized in Table 1 including corresponding data from the historical cohort (11, 12). Patients treated with the Stupp's regimen in 2014–2017 were older than the historical cohort (*p* = 0.034) but underwent more often radical resection (*p* < 0.001). Postsurgery MRI exam was performed in 97 (63%) patients and was more common in patients after GTR or subtotal resection (STR).

**Table 1:** Basic patients' characteristics of current cohort (GBM 2014–2017) and historical group (GBM 2003–2009).

**Study cohort**	**GBM 2014–2017 (*****n*** **=** **155)**		**GBM 2003–2009 (*****n*** **=** **145)**		**Current vs. historical group**
	**Stupp's regimen *n* = 90**	**RT alone *n* = 65**	**Stupp's regimen *n* = 86**	**RT alone *n* = 59**	Stupp's regimen *p*-value^*^
**Age (years)**					
Median (IQR)	56 (30-76)	66 (20-86)	56 (24-69)	67 (41–82)	**0.034**
≤50	22 (24%)	10 (15%)	30 (35%)	5 (8%)	0.140
**Mens**	61 (68%)	34 (52%)	51 (59%)	33 (56%)	0.274
**Performance status (ECOG) and Karnofsky index (KI)**					0.450
ECOG 0 (KI 90–100%)	45 (50%)	11 (17%)	38 (44%)	6 (10%)	
ECOG 1 (KI 70–80%)	44 (49%)	38 (58%)	48 (56%)	35 (59%)	
ECOG 2 (KI 50–60%)	1 (1%)	16 (25%)	0 (0%)	18 (31%)	
**Tumor location**					
Deep brain location	23 (26%)	26 (40%)	NA	NA	
**Extent of resection**					**<0.001**
GTR	44 (49%)	14 (22%)	17 (20%)	8 (13%)	
STR	36 (40%)	24 (37%)	56 (65%)	21 (36%)	
Partial resection or biopsy	10 (11%)	27 (41%)	13 (15%)	30 (51%)	
**IDH status**					
Mutated/evaluated	5/57 (9%)	1/22 (5%)	NA	NA	
**MGMT status**					
Metylated/evaluated	11/48 (23%)	8/25 (32%)	12/38 (32%)	NA	

GBM, glioblastoma; CHT/RT, chemoradiotherapy; CHT, chemotherapy; RT, radiotherapy; NA, Not Available; ECOG, Eastern Cooperative Oncology Group; GTR, gross total resection; STR, subtotal resection; IDH, Isocitrate dehydrogenase; MGMT, O6-methylguanine-DNA-methyltransferase.

* *p-values < 0.05 are marked in bold*.

The median time to first RT session was 6.7 weeks (range 2.1–11.7 weeks). The majority of patients (91%) were irradiated by intensity-modulated radiotherapy technique (including arc therapy—volumetric modulated RT). Among patients who were not indicated to Stupp's regimen, the most common abbreviated schedule was 15 × 2.67 Gy (11/65; 17%) and 20 × 2.5 Gy (17/65; 26%). Stupp's regimen was indicated in 90/155 patients (58%). Only 31/90 (34%) patients finished the whole Stupp's protocol with all six cycles of adjuvant chemotherapy, 47/90 (52%) finished at least three cycles. More details about patients' treatment are summarized in Table 2.

**Table 2:** Patients' treatment.

**Study cohort**	**GBM 2014–2017 (*****n*** **=** **155)**		**GBM 2003–2009 (*****n*** **=** **145)**		**Current vs. historical group**
	**Stupp's regimen *n* = 90**	**RT alone *n* = 65**	**Stupp's regimen *n* = 86**	**RT alone *n* = 59**	Stupp's regimen *p*-value^*^
**Time to RT initiation**					
Median (weeks)	6.7	6.9	5.1	5.3	**<0.001**
>6 weeks	57 (63%)	43 (66%)	27 (31%)	22 (39%)	**<0.001**
**Radiotherapy**					
Median dose	60	40	60	50	0.430
Abbreviated RT 15 × 2.67 Gy	0	11/65 (17%)	0	0	
Abbreviated RT 20 × 2.5 Gy	0	17/65 (26%)	0	9/59 (15%)	
Contouring approach EORTC	32 (36%)	53 (82%)	NA	NA	
Contouring approach RTOG	58 (64%)	5 (8%)	NA	NA	
**Chemoradiotherapy**					
Duration (days; IQR)	42 (37–44)	0	42	0	
Corticosteroids use	53/86 (62%)	57/62 (92%)	NA	NA	
**Adjuvant chemotherapy**					
No. of patients	65/90 (72%)	0	34/86 (40%)	0	**<0.001**
No. of cycles: median (range)	4 (1–15)	0	4 (1–12)	0	
No. of cycles: ≥3	47/90 (52%)		26/86 (30%)	0	
No. of cycles: ≥6	31/90 (34%)	0	11/86 (13%)	0	
**Treatment after progression**					
No. of patients	47/79 (59%)	9/32 (28%)	46/67 (69%)	NA	
Surgery	20/47 (43%)	1/9 (11%)	21/46 (46%)	NA	
Chemotherapy	33/47 (70%)	8/9 (89%)	39/46 (85%)	NA	
Reirradiation	20/47 (43%)	1/9 (11%)	8/46 (17%)	NA	

GBM, glioblastoma; CHT/RT, chemoradiotherapy; CHT, chemotherapy; RT, radiotherapy; NA, Not Available.

* *p-values < 0.05 are marked in bold*.

**Figure 1:**
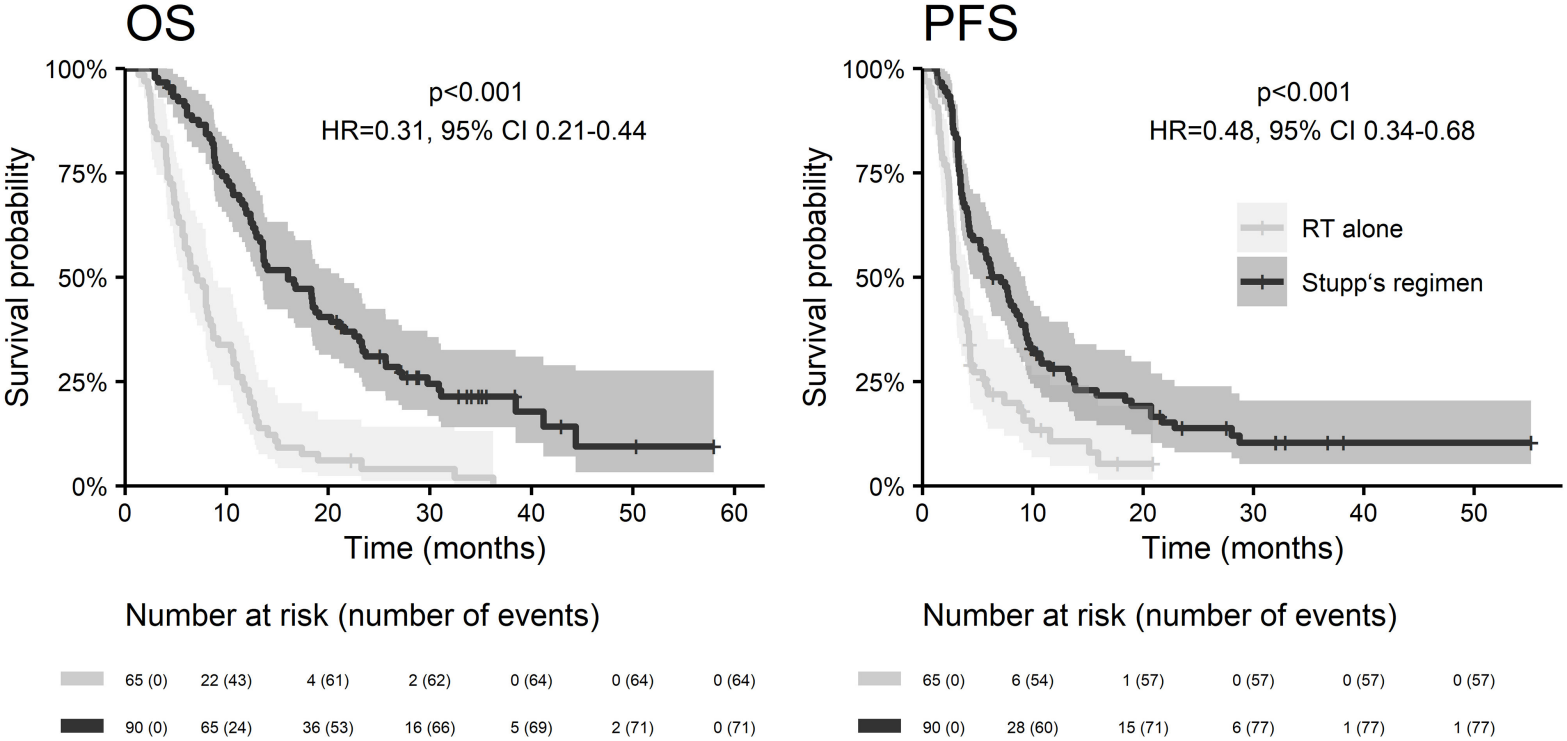
OS and PFS according to Stupp's regimen indication.

**Figure 2:**
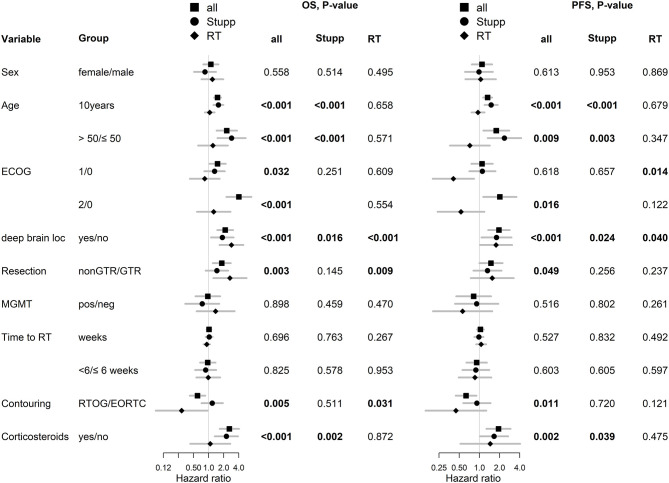
Univariable analysis for OS and PFS in whole, Stupp's regimen cohort, and RT only cohorts. p-values < 0.05 are marked in bold.

**Figure 3:**
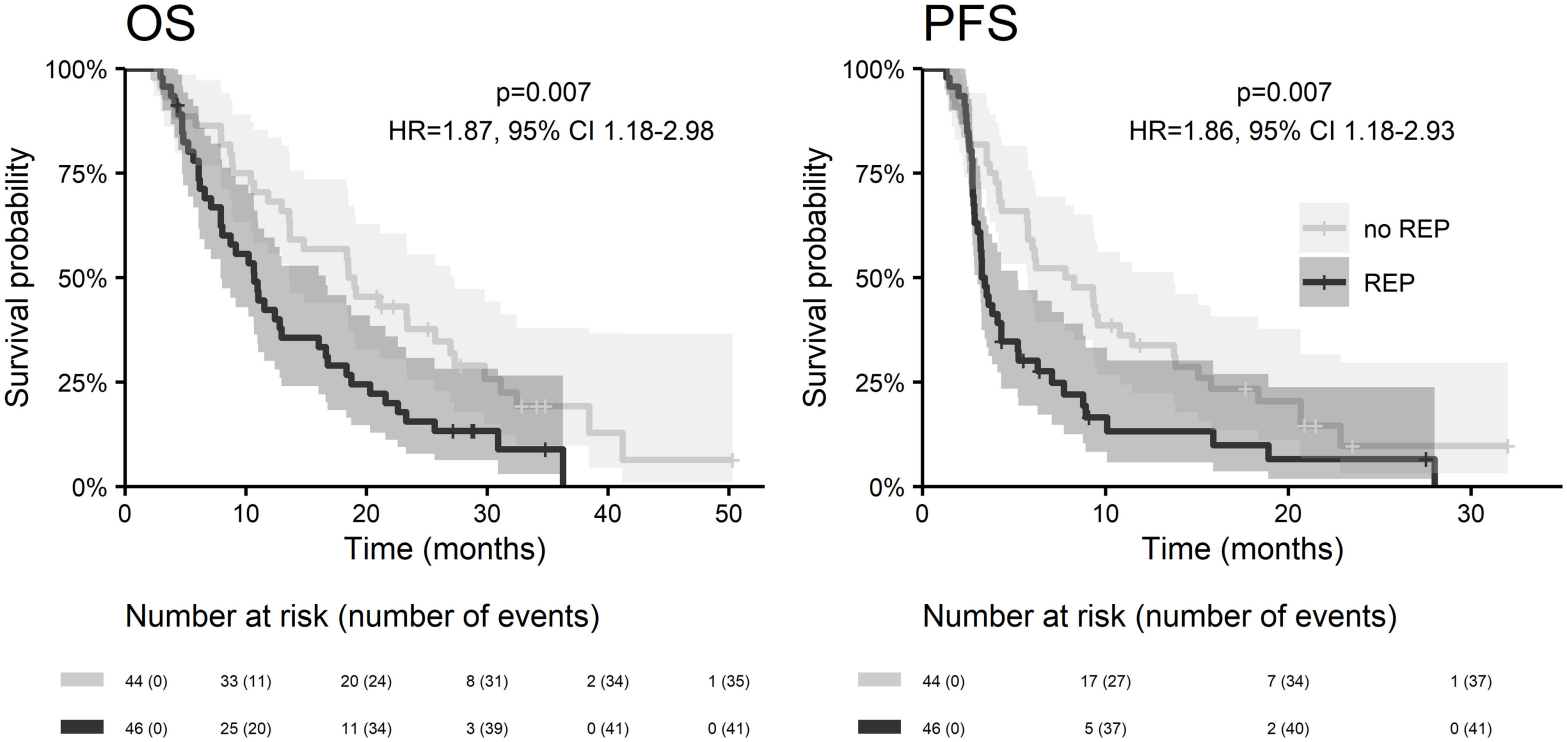
OS and PFS in the context of a presence of rapid early progression on planning MRI.

With a median follow up of 34.8 months, the median PFS for the whole study cohort was 4.2 months and 2-year PFS 10%. Corresponding values for OS were 11.6 months and 19.8%. Treatment with Stupp's regimen was a strong positive prognostic factor with HR 0.31 (*p* < 0.001, median 16.0 vs. 7.1 months) and HR 0.48 (*p* < 0.001, median 6.7 vs. 3.1 months) for OS and PFS, respectively (Figure 1). Univariable analysis of prognostic factors for OS and PFS in the whole study cohort, Stupp's regimen cohort, and radiotherapy alone cohort is summarized in Figure 2 and Table 3. In the whole cohort, the median OS of patients over 50 years was significantly shorter than that of younger patients (10.7 vs. 20.2 months; HR 2.31; *p* < 0.001). Better OS was observed in patients after GTR (median 15.4 vs. 11.8 months; HR 0.54; *p* = 0.003), those with better ECOG score (median 13.6 vs. 10.3 vs. 5.8 months for EOCG 0, 1, 2 respectively; *p* < 0.001), patients with contouring based on RTOG approach (median 14.0 vs. 10.7 months; HR 0.60; p = 0.005) and patients without corticosteroids, as well as without deep brain tumor location (related to possibility to achieve GTR). No difference in OS and PFS was observed in our considered cohorts with respect to MGMT methylation status. The interval between surgery and the start of radiotherapy (median 6.7 weeks) was not prognostically significant (*p* = 0.825 and 0.603 for OS and PFS, respectively). On the other hand, the presence of rapid early progression on planning MRI (in 46 patients out of 90 evaluable patients who had postsurgery MRI) was associated with significantly worse survival (median 10.7 vs. 18.7 months; HR 1.87; *p* = 0.007, 2-year OS 15.6 vs. 37.7%), Figure 3.

**Table 3:** Univariable analysis for OS and PFS in whole, Stupp's regimen, and RT only cohorts.

		**OS**					
		**All**		**Stupp's regimen**		**RT alone**	
**Variable**	**Group**	**HR (95% CI)**	*P*-value^*^	**HR (95% CI)**	*P*-value^*^	**HR (95% CI)**	*P*-value^*^
Sex	Female/male	1.11 (0.78–1.57)	0.558	0.85 (0.51–1.40)	0.514	1.19 (0.72–1.97)	0.495
Age	10 years	1.51 (1.28–1.76)	**<0.001**	1.57 (1.25–1.97)	**<0.001**	1.05 (0.84–1.31)	0.658
	>50/≤50	2.31 (1.44–3.69)	**<0.001**	2.87 (1.50–5.48)	**<0.001**	1.22 (0.61–2.42)	0.571
ECOG	1/0	1.51 (1.04–2.19)	**0.032**	1.32 (0.82–2.11)	0.251	0.84 (0.42–1.66)	0.609
	2/0	4.02 (2.27–7.14)	**<0.001**	NA	NA	1.26 (0.58–2.73)	0.554
Deep brain loc	Yes/no	2.15 (1.49–3.08)	**<0.001**	1.88 (1.12–3.15)	**0.016**	2.85 (1.65–4.92)	**<0.001**
Resection	Non-GTR/GTR	1.84 (1.22–2.77)	**0.003**	1.46 (0.87–2.45)	0.145	2.66 (1.24–5.72)	**0.009**
MGMT	Pos/neg	0.96 (0.55–1.70)	0.898	0.74 (0.34–1.63)	0.459	1.38 (0.58–3.30)	0.470
Time to RT	Weeks	1.02 (0.92–1.14)	0.696	1.02 (0.87–1.20)	0.763	0.92 (0.79–1.07)	0.267
	<6/≤6 weeks	0.96 (0.67–1.37)	0.825	0.87 (0.54–1.41)	0.578	0.98 (0.57–1.70)	0.953
Contouring	RTOG/EORTC	0.60 (0.42–0.86)	**0.005**	1.18 (0.72–1.93)	0.511	0.29 (0.09–0.95)	**0.031**
Corticosteroids	Yes/no	2.60 (1.67–4.05)	**<0.001**	2.26 (1.33–3.85)	**0.002**	1.08 (0.43–2.71)	0.872
		**PFS**					
Sex	Female/male	1.09 (0.77–1.55)	0.613	0.99 (0.61–1.59)	0.953	1.04 (0.62–1.76)	0.869
Age	10 years	1.33 (1.14–1.55)	**<0.001**	1.50 (1.19–1.88)	**<0.001**	0.95 (0.76–1.19)	0.679
	>50/≤50	1.79 (1.15–2.79)	**0.009**	2.36 (1.33–4.20)	**0.003**	0.72 (0.36–1.43)	0.347
ECOG	1/0	1.10 (0.76–1.58)	0.618	1.11 (0.71–1.74)	0.657	0.41 (0.20–0.83)	**0.014**
	2/0	2.02 (1.14–3.57)	**0.016**	NA	NA	0.53 (0.24–1.18)	0.122
Deep brain loc	Yes/no	1.96 (1.35–2.83)	**<0.001**	1.80 (1.07–3.01)	**0.024**	1.77 (1.02–3.08)	**0.040**
Resection	Non-GTR/GTR	1.49 (1.00–2.22)	**0.049**	1.32 (0.82–2.14)	0.256	1.56 (0.74–3.25)	0.237
MGMT	Pos/neg	0.82 (0.45–1.49)	0.516	0.91 (0.43–1.91)	0.802	0.56 (0.20–1.55)	0.261
Time to RT	Weeks	1.04 (0.92–1.17)	0.527	0.98 (0.84–1.15)	0.832	1.06 (0.89–1.26)	0.492
	<6/≤6 weeks	0.91 (0.64–1.30)	0.603	0.88 (0.56–1.41)	0.605	0.86 (0.49–1.51)	0.597
Contouring	RTOG/EORTC	0.63 (0.44–0.90)	**0.011**	0.92 (0.57–1.47)	0.720	0.45 (0.16–1.27)	0.121
Corticosteroids	Yes/no	1.93 (1.28–2.92)	**0.002**	1.66 (1.02–2.68)	**0.039**	1.45 (0.52–4.05)	0.475

OS, overall survival; PFS, progression free survival; RT, radiotherapy; NA, Not applicable; ECOG, Eastern Cooperative Oncology Group; GTR, gross total resection; MGMT, O6-methylguanine-DNA-methyltransferase.

* *p-values < 0.05 are marked in bold*.

**Figure 4:**
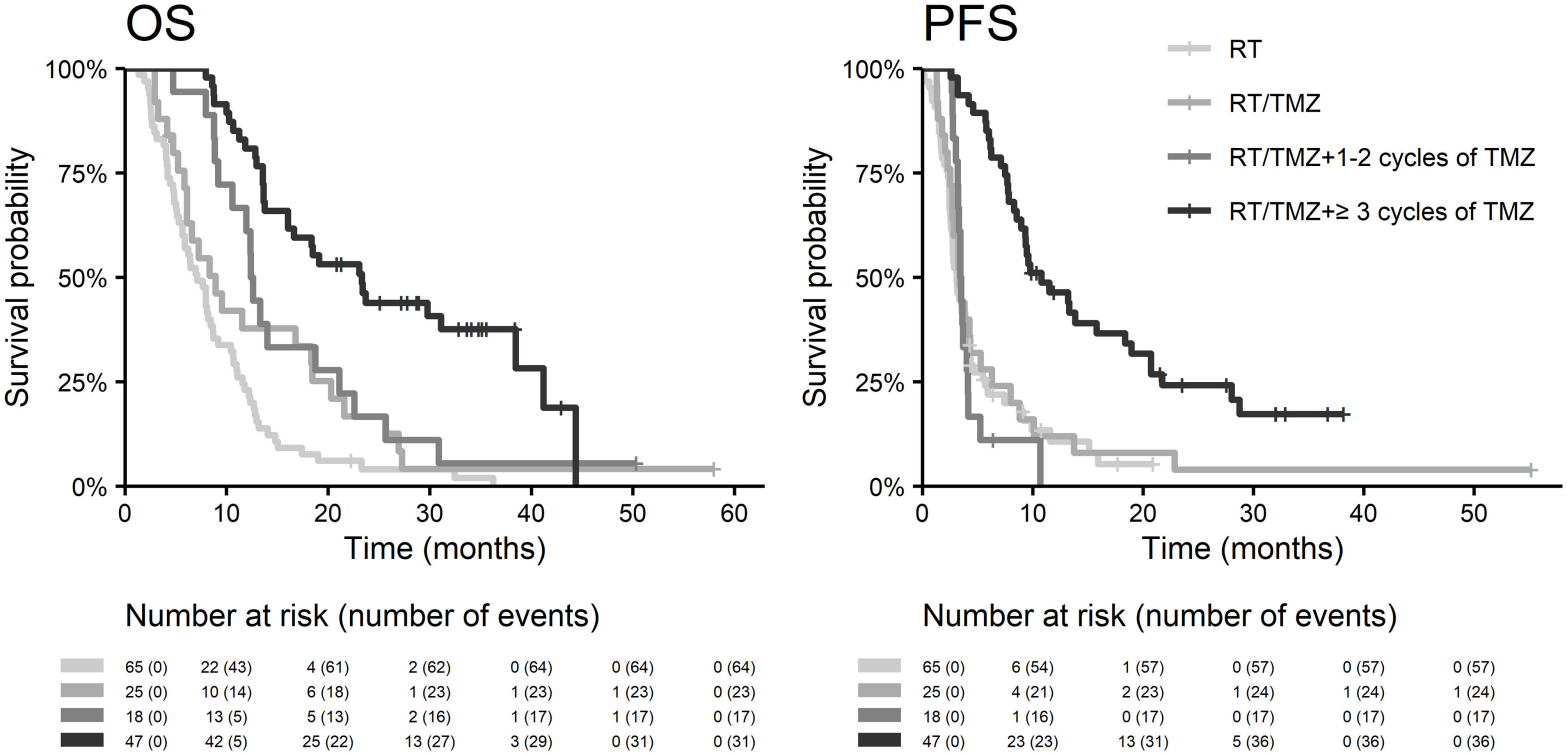
OS and PFS in the context of adjuvant chemotherapy.

**Figure 5:**
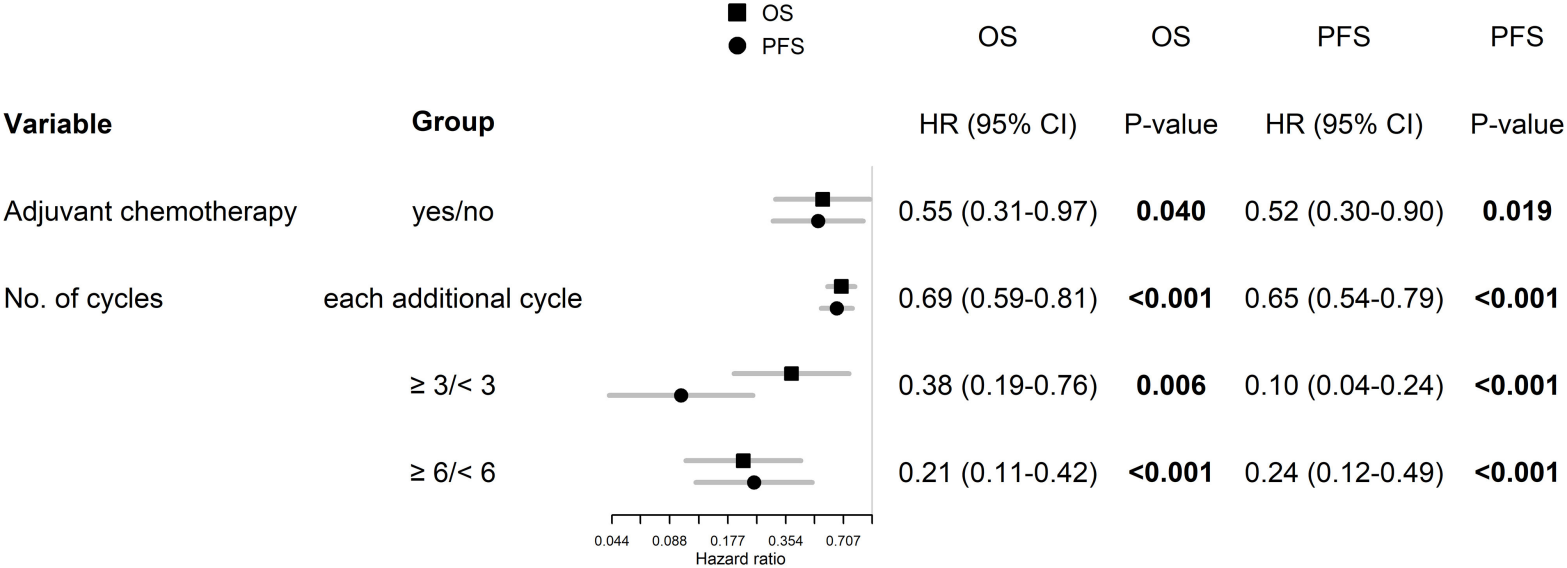
Age, extent of resection, and ECOG patient status adjusted hazard ratios for patients, who were able to continue in adjuvant chemotherapy.

The best outcomes had patients, who were able to continue in adjuvant chemotherapy after chemoradiotherapy (median OS 23.3 months and 2-year survival of 43.9% in those who finished at least three cycles of adjuvant chemotherapy), Figure 4. Survival outcome was associated with adjuvant chemotherapy also after adjusting for age, extent of resection, and ECOG patient status (Figure 5).

**Figure 6:**
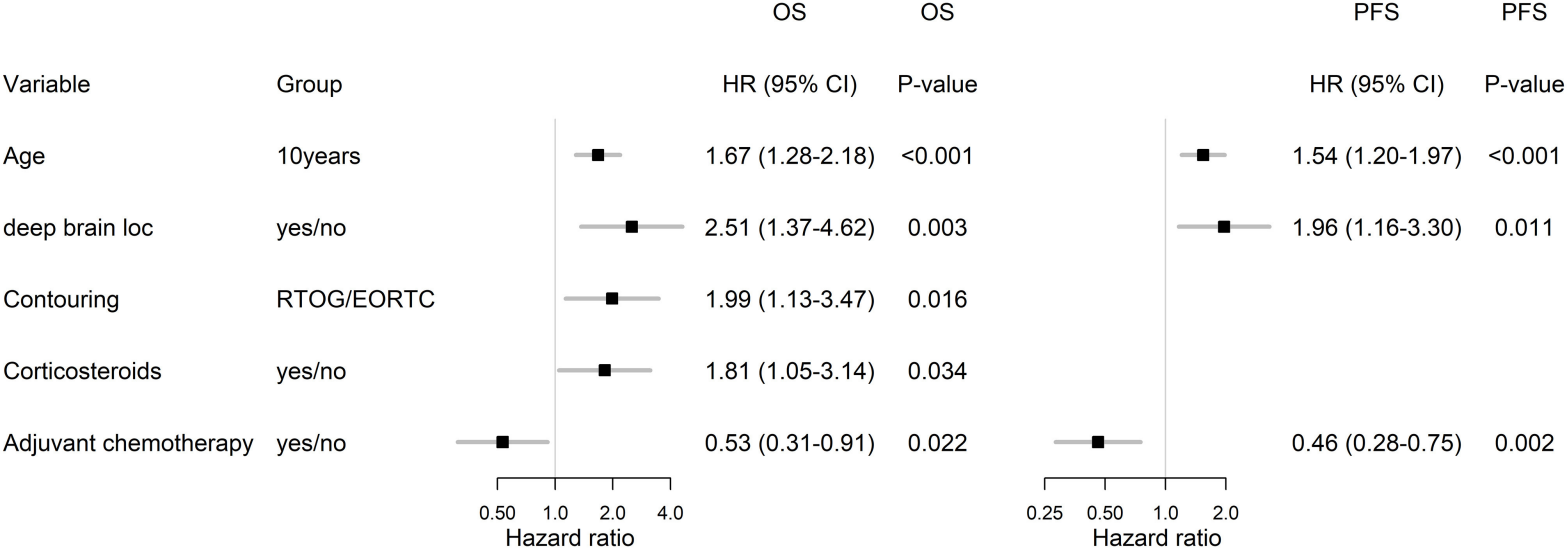
Multivariable analysis for OS and PFS in Stupp's regimen cohort.

In the subgroup of patients treated by Stupp's protocol, the age, deep brain tumor location, contouring approach, corticosteroids, and adjuvant chemotherapy were independently associated with OS and age, deep brain tumor location, and adjuvant chemotherapy were independently associated with PFS (Figure 6).

In comparison with 86 patients from historical control treated by “the same” Stupp's regimen, the positive trend in the increase of overall survival was observed (median OS 13.8 vs. 16.0 months), Table 4 (11). The hazard ratios (Stupp's regimen vs. RT alone) were adjusted for age, extent of resection, and ECOG patient status due to comparability with historical results (historical cohort and original Stupp's trial) (1, 11). Adjusted HRs in current cohort were 0.45 (95% CI: 0.27–0.75, *p* = 0.002) and 0.55 (95% CI: 0.32–0.93, *p* = 0. 025) for OS and PFS, respectively.

**Table 4:** Survival outcomes (months) in comparison with previous cohorts.

	**CHT/RT (MMCI 2014–2017) *n* = 90**	**CHT/RT (MMCI 2003–2009) *n* = 86**	**CHT/RT [Stupp trial (1)] *n* = 287**
Median follow up (months)	34.8	NA	28
**Overall survival**			
Median	16.0	13.8	14.6
1-year	65%	58%	61%
2-year	31%	28%	27%
3-year	21%	7%	16%
4-year	10%	2%	12%
5-year	NA	2%	10%
**Progression free survival**			
Median	6.7	7.8	6.9
1-year	28%	32%	27%
2-year	14%	9%	11%

*OS, overall survival; PFS, progression free survival; CHT/RT, chemoradiotherapy; MMCI, Masaryk Memorial Cancer Institute; NA, Not Available*.

## Discussion

Remarkable improvement in overall survival after Stupp's regimen treatment was observed in recent years in comparison to the historical cohort treated a decade ago in our cancer center. Using the same treatment protocol in daily real-world practice, we observed improvement in median OS more than 2 months, with similar median PFS. Absence in PFS improvement may probably be due to the frequent unavailability of postoperative MRI examination 10 years ago. In the cohort of subsequently treated patients according to Stupp's regimen, postoperative MRI was performed in only 20% (17/86) of patients. The evaluation of the finding on the first MRI examination after completed radiotherapy was then very problematic. Progression was often closed according to the second MRI examination. Similar improvement in OS through decade was also observed in reports from recent prospective randomized clinical trials where treatment in control study arm usually consisted of Stupp's regimen. However, care must be taken in comparison of survival data with respect to time of randomization (some of the recent clinical trials randomized patients after the end of chemoradiotherapy phase) (10, 13, 14). In the ACT IV study with rindpopepimut, the median OS with Stupp's regimen alone (control arm) was 20.2 months (median 2.8 months from diagnosis to randomization + median 17.4 months from randomization to death) and in EF-14 with Optune median OS 19.8 months as discussed below (13, 14). Randomization in the EF-14 study (Stupp's regimen + Optune vs. Stupp's regimen alone) occurred after chemoradiotherapy was finished and only patients without progression of the disease were enrolled (92%), the remaining 8% of patients were excluded. Hypothetically, if the same proportion of patients with the worst prognosis (8%) were excluded from the Stupp's study EORTC 26981–22981/NCIC CE3, then the median OS of patients treated with Stupp's regimen would increase to around 16.5 months from randomization and to around 17.7 months from diagnosis. The difference in overall survival of 2–3 months is probably due to advances in diagnostic and treatment methods (20.2 months in ACT IV and 19.8 months in EF-14 vs. hypothetical 17.7 months in the Stupp's study) (10, 13, 14). The same improvement was also observed in our real-world cohort of patients treated outside of clinical trials.

The original Stupp's regimen (EORTC 26981–22981/NCIC CE3) was published already in 2005 and represents one of the most influencing prospective clinical trial in general (1). Indeed, the referred paper is unequivocally the most cited one (Publication Year 2005) in premium *the New England Journal of Medicine* journal. Only a few subsequent reports indicate so far possible improvements, mainly based on trials focused on alternative temozolomide scheduling, as is prolonged administration to 12 or even more months (19). However, based on a meta-analysis of 4 randomized clinical trials comparing outcomes after six vs. more cycles adjuvant chemotherapy, no difference in OS was observed next to only slight improvement in PFS (19). Moreover, not every patient is actually able to finish in daily clinical practice the predefined 6 months of adjuvant treatment due to worsening clinical status or already progressing tumor. In our recent cohort, 52% (47/90) of patients were able to finish three and more cycles, and only 34% (31/90) completed six cycles of temozolomide (in original Stupp's cohort 47% of patients finished six cycles).

Neither other modern targeted therapies (bevacizumab, anti-EGFR inhibitors and antibodies, integrin inhibitors, anti-EGFR antibody conjugate, and depatuxizumab mafodotin cytostatics) have been able to improve the Stupp regimen (20–26). Similarly, immunotherapy with rindopepimut or the anti-PD-1 antibody nivolumab, which has been currently successful in a number of poorly treatable diagnoses, has not been successful (13).

The only positive phase 3 clinical trial since 2005 that partially changed the glimpse of the treatment standard of newly diagnosed glioblastomas is the EF-14 study with Optune. The principle of treatment is based on the application of alternating current via Tumor Treating Fields to the tumor by means of electrodes adhered to the scalp, which prevent tumor cell mitosis (14, 27). Since treatment with TTF is not appropriate/accepted by and also available to every patient with glioblastoma, the Stupp's regimen should still be considered the gold standard (28). Similarly, intensification of chemotherapy in patients with MGMT methylation (lomustine + temozolomide combination) will probably not be a major breakthrough. The scheme is accepted with embarrassment, mainly because of concerns about a potential increase in toxicity (29).

The essential prerequisite for further optimization of treatment in daily clinical practice is the auto evaluation of own cohorts with comparison to published guidelines defining clinical trials. We performed the first major analysis of glioblastoma patients treated by the Stupp's regimen in 2011. At that time, patients treated in our cancer center achieved a similar median overall survival (13.8 vs. 14.6 months), the same 2-year survival after surgery (28 vs. 27%), but lower 5-year survival (2 vs. 10%) compared to outcomes from original Stupp et al. trial (Table 4) (1, 11). In the recent evaluation of patients treated in 2014–2017, we confirmed the importance of known prognostic factors (except for MGMT methylation) and compared the overall survival to patients treated previously in 2003–2009 (86 patients). Similar to improvements observed in mentioned clinical trials, we also described increased survival in our cohort of patients treated outside of clinical trials (median overall survival increased from 13.8 to 16.0 months, the 2-year survival rate increased from 28 to 31% and 4-year survival increased from 2 to 10%), Table 4. This improvement in OS is also reflected by better adjusted HR for treatment by Stupp regimen HR 0.45 (95% CI: 0.27–0.75, *p* = 0.002) in comparison to that reported in original Stupp paper [HR 0.63 (95% CI: 0.52–0.75) *p* < 0.001]. However, it should be acknowledged that the proportion of patients treated with adjuvant temozolomide increased significantly, mainly thanks to better toxicity management and improvements in general comprehensive cancer care. In the historical cohort, adjuvant chemotherapy after chemoradiotherapy was indicated in only 40% of patients, whereas in the current cohort it was already 72% (65/90), which is close to that in the Stupp and colleagues trial (78%) (1, 11). In addition, a subanalysis of 47 patients who underwent three or more cycles of adjuvant temozolomide revealed a significant increase in overall survival to 23.3 months and nearly 44% of patients achieved 2-year OS. From these results, it is evident that the continuation of adjuvant temozolomide is crucial. Premature discontinuation of chemotherapy should be avoided due to unclear findings at the first MRI follow-up after chemoradiotherapy (30–34). Attention should be paid to the differential diagnosis of pseudoprogression, with cooperation of radiation oncologist and neuroradiologist (35) as well as with employment of advanced imaging methods. Definitely, some patients (including those in original Stupp and colleagues trial) developed pseudoprogression, and wrong discontinuation of chemotherapy affected their survival. We can only assume the same bias rate in both historical and current cohort and thus relatively low influence on the overall survival analysis.

In the current analysis, we also addressed the issue of rapid early recurrence at planning MRI and its effect on overall survival (evaluated strictly only in patients who underwent postoperative MRI). The incidence of rapid early progression was 51% high, what incidence is in accordance with recent publications (36–39). We confirmed its significant negative prognostic effect on overall survival and progression-free survival (Figure 3). More aggressive treatment of this especially risky group of patients warrants further interest in future clinical trials.

The inherent limitation of this study is its retrospective nature. On the other hand, the methodology to obtain data describing the truly real clinical experience must be retrospective in nature. Thus, this represents both the strength as well as the limitation of this single institution study unbiased by inter-center variability. Definitely, there are enormous unmeasurable biases in the way the patients were treated during a decade (extent of surgery, demographic features, etc.) and these are likely different in the two time cohorts. Even more biases would be in the case we would aim to compare results with original trial by Stupp et al. (1) (patients enrollment between 2000 and 2002; patients enrolled in many institutions in many countries; ability to recover after brain surgery and ability to achieve better performance status also thanks to safer neurosurgery). For these reasons, we focused mainly on a comparison of our own two cohorts, and mentioned differences were acknowledged in statistical methodology. Some results (for example effect of RT target volumes contouring strategy on OS and observation of association between OS and number of temozolomide cycles in the adjuvant phase) warrant further detailed evaluation.

## Conclusions

Age, performance status, extent of resection, the presence of rapid early recurrence before radiotherapy, MGMT gene promoter methylation, ability to finish concomitant chemoradiotherapy, and adjuvant chemotherapy significantly influence the prognosis of glioblastoma patients. According to an analysis of a recent group of patients treated outside of clinical trials with the Stupp's regimen, we have shown a clear trend in extending overall survival over the last decade, despite the absence of a new treatment method. An important factor is the completion of the full Stupp's regimen. The most important is multidisciplinary cooperation and medical progress in both the area of diagnostics and individual treatment methods.

## Data Availability Statement

The raw data supporting the conclusions of this article will be made available by the authors, without undue reservation.

## Ethics Statement

The studies involving human participants were reviewed and approved by Ethical Committee, Masaryk Memorial Cancer Institute. Written informed consent to participate in this study was provided by the participants' legal guardian/next of kin.

## Author Contributions

Conceptualization: TK, MS, RJ, JS, OS, and PS. Data curation: IS, PP, PF, VV, and OS. Formal analysis: RL, TK, AP, JS, and LH. Funding acquisition: RL, TK, and MH. Investigation: RL, TK, RB, PF, RJ, KM, MH, JS, and OS. Methodology: RL, TK, IS, RB, and JS. Project administration: LH. Supervision: PS. Validation: IS, AP, and PP. Writing–original draft: RL, TK, and IS. Writing–review and editing: TK, MS, OS, and PS. All authors contributed to the article and approved the submitted version.

## Conflict of Interest

The authors declare that the research was conducted in the absence of any commercial or financial relationships that could be construed as a potential conflict of interest.
